# Brain–Blood Partition Coefficient and Cerebral Blood Flow in Canines Using Calibrated Short TR Recovery (CaSTRR) Correction Method

**DOI:** 10.3389/fnins.2019.01189

**Published:** 2019-11-05

**Authors:** Scott W. Thalman, David K. Powell, Margo Ubele, Christopher M. Norris, Elizabeth Head, Ai-Ling Lin

**Affiliations:** ^1^F. Joseph Halcomb III, Department of Biomedical Engineering, University of Kentucky, Lexington, KY, United States; ^2^Sanders–Brown Center on Aging, University of Kentucky, Lexington, KY, United States; ^3^Magnetic Resonance Imaging and Spectroscopy Center, University of Kentucky, Lexington, KY, United States; ^4^Department of Pharmacology and Nutritional Sciences, University of Kentucky, Lexington, KY, United States; ^5^Department of Pathology and Laboratory Medicine, University of California, Irvine, Irvine, CA, United States; ^6^University of California Irvine Institute for Memory Impairments and Neurological Disorders (UCI MIND), University of California, Irvine, Irvine, CA, United States; ^7^Department of Neuroscience, University of Kentucky, Lexington, KY, United States

**Keywords:** cerebral blood flow, brain–blood partition coefficient, calibrated short TR recovery, arterial spin labeling, perfusion, magnetic resonance imaging, canines

## Abstract

The brain–blood partition coefficient (BBPC) is necessary for quantifying cerebral blood flow (CBF) when using tracer based techniques like arterial spin labeling (ASL). A recent improvement to traditional MRI measurements of BBPC, called calibrated short TR recovery (CaSTRR), has demonstrated a significant reduction in acquisition time for BBPC maps in mice. In this study CaSTRR is applied to a cohort of healthy canines (*n* = 17, age = 5.0 – 8.0 years) using a protocol suited for application in humans at 3T. The imaging protocol included CaSTRR for BBPC maps, pseudo-continuous ASL for CBF maps, and high resolution anatomical images. The standard CaSTRR method of normalizing BBPC to gadolinium-doped deuterium oxide phantoms was also compared to normalization using hematocrit (Hct) as a proxy value for blood water content. The results show that CaSTRR is able to produce high quality BBPC maps with a 4 min acquisition time. The BBPC maps demonstrate significantly higher BBPC in gray matter (0.83 ± 0.05 mL/g) than in white matter (0.78 ± 0.04 mL/g, *p* = 0.006). Maps of CBF acquired with pCASL demonstrate a negative correlation between gray matter perfusion and age (*p* = 0.003). Voxel-wise correction for BBPC is also shown to improve contrast to noise ratio between gray and white matter in CBF maps. A novel aspect of the study was to show that that BBPC measurements can be calculated based on the known Hct of the blood sample placed in scanner. We found a strong correlation (*R*^2^ = 0.81 in gray matter, *R*^2^ = 0.59 in white matter) established between BBPC maps normalized to the doped phantoms and BBPC maps normalized using Hct. This obviates the need for doped water phantoms which simplifies both the acquisition protocol and the post-processing methods. Together this suggests that CaSTRR represents a feasible, rapid method to account for BBPC variability when quantifying CBF. As canines have been used widely for aging and Alzheimer’s disease studies, the CaSTRR method established in the animals may further improve CBF measurements and advance our understanding of cerebrovascular changes in aging and neurodegeneration.

## Introduction

When using tracer-based techniques like arterial spin labeling (ASL) to quantify cerebral blood flow (CBF), it is necessary to determine the partition coefficient of the tracer between the perfused tissue and the arterial blood. ASL is a non-invasive, quantitative magnetic resonance imaging (MRI) technique that uses magnetically labeled protons in the water molecules of the blood as the tracer (Williams et al., 1992; Petcharunpaisan et al., 2010; Alsop et al., 2015). So in the case of ASL, the relevant partition coefficient is the brain–blood partition coefficient of water (BBPC) which is the ratio of the solubility of water in brain tissue to the solubility of water in the blood. The BBPC is tissue specific and varies with age, species, pathology, and brain region (Herscovitch and Raichle, 1985; Kudomi et al., 2005; Leithner et al., 2010; Hirata et al., 2011). This means that BBPC should be determined experimentally for each subject.

However, the standard practice in ASL studies is to assume a constant BBPC value of 0.9 mL/g for all regions of the brain regardless of the known variability of this parameter (Herscovitch and Raichle, 1985; Alsop et al., 2015). This assumption is made because the previously published MRI methods to experimentally determine BBPC required prohibitively long acquisition times and ASL studies were generally focused on gray matter perfusion where BBPC variability was assumed to be small (Roberts et al., 1996; Leithner et al., 2010). A recent study in mice at 7T reported an 87% reduction in the acquisition time for BBPC maps using an MRI technique called calibrated short TR recovery (CaSTRR) (Thalman et al., 2019). Like previous methods, CaSTRR determines relative proton density using a series of gradient echo acquisitions with varying repetition times (TR) and then calibrates the proton density map using a set of deuterium doped phantoms which provide an absolute scale of water content. The method is accelerated in CaSTRR by using shorter TR values and using gadolinium doped water phantoms to acquire similar quality BBPC maps in a fraction of the time.

The goal of this study is to apply the CaSTRR technique to a cohort of healthy canines using a protocol suited for application in humans at 3T. To do so we acquired BBPC images using a CaSTRR protocol adapted for use on a 3T human scanner. We then acquired CBF maps using pseudo-continuous ASL (pCASL) to assess the effect of BBPC correction on CBF maps, and high resolution anatomical images using magnetization prepared rapid acquisition gradient echo (MPRAGE) to facilitate segmentation and coregistration. Finally, we compare two methods of normalizing the proton density maps using the doped water phantoms and using blood water content derived from Hct values.

## Materials and Methods

### Animals

All animal experiments were performed in accordance with NIH guidelines and approved by the University of Kentucky Institutional Animal Care and Use Committee (approval number #2017–2680). Middle aged beagles (*n* = 17, age = 5.0–8.0 years, male = 24%) were acquired as part of a longitudinal study on aging and Alzheimer’s disease. The scans in this report represent pretreatment observations, and all animals were healthy at the time of their scans. The animals were anesthetized during the MRI procedure using 3–4 mg/Kg propofol for induction and 1–4% isoflurane mixed with air for maintenance. Respiratory rate, heart rate, body temperature, and blood pressure were monitored and maintained throughout the procedure. Two 5 mL vials of blood were drawn from the jugular vein using ethylenediaminetetraacetate (EDTA) treated vials. One of these was placed in the scanner with the animal according to the CaSTRR protocol, and the other was sent for laboratory analysis including Hct (ANTECH Diagnostics, Louisville, KY, United States).

### Scanning Procedure

Magnetic resonance imaging experiments were performed using a 3T Siemens Prisma scanner (Siemens, Erlangen, Germany) at the MRI and Spectroscopy Center at the University of Kentucky. The animal was placed prone with their head resting in a 155 mm diameter, 15 channel transmit/receive birdcage coil commonly used for scanning human knees. The doped water phantoms along with blood sample were centered on the top of the head. The CaSTRR, pCASL, and MPRAGE acquisitions were all acquired in a single scanning session.

### Calibrated Short TR Recovery Imaging

**FIGURE 1:**
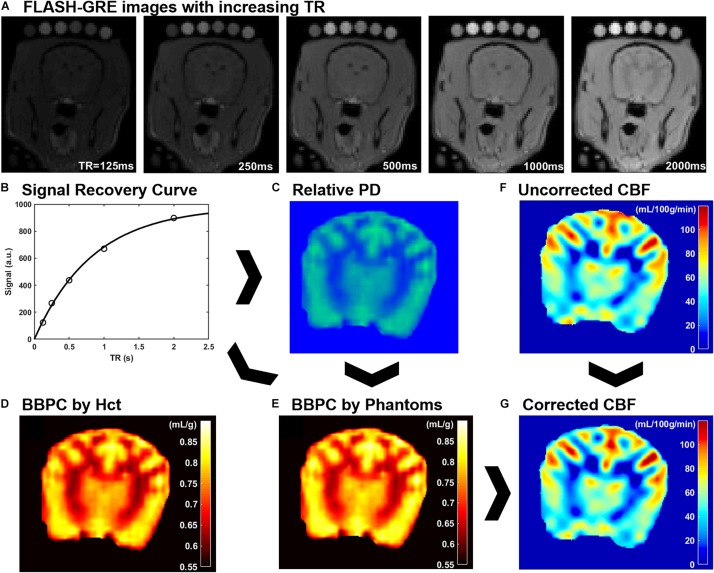
An explanation of CaSTRR and pCASL methods used in this study. CaSTRR utilizes a series of FLASH-GRE acquisitions with varying TR which include a blood sample and gadolinium-doped deuterium samples placed on the head **(A)**. An exponential regression is fit to the signal recovery curve for each voxel **(B)** yielding a map of relative proton density values **(C)**. The relative proton density values are calibrated using either water content estimated from hematocrit (Hct) **(D)** or the scale of water content present in the phantoms **(E)**. Uncorrected CBF maps are derived from pCASL acquisitions **(F)** and are corrected on a voxel-wise basis using the BBPC map normalized to the phantoms to create a corrected map of CBF **(G)**.

For the CaSTRR proton density measurements a series of 2-D image stacks were acquired using a phase-spoiled, fast low-angle shot gradient echo (FLASH-GRE) sequence with varying repetition times (TR = 125, 250, 500, 1000, and 2000 ms) (Thalman et al., 2019). The shortest possible echo time (TE = 1.9 ms) was used to minimize T2^∗^ decay. Image matrix parameters were: field of view = 135 × 124 mm, matrix = 96 × 88, in-plane resolution = 1.4 × 1.4 mm, slice thickness = 3 mm, number of slices = 30, flip angle = 90°, acquisition time = 4 min, labeling offset = 12 mm (see Figure 1A). A B_1_ mapping was done to confirm accuracy and homogeneity of the B_1_ field. A simulation with a saline phantom was used to simulate the gradient echo signal over a range of flip angles (see Supplementary Figure S1).

**FIGURE 2:**
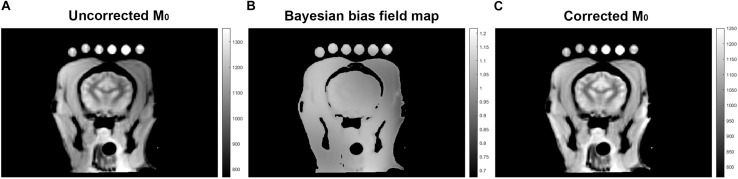
A representative images of M_0_ as calculated by exponential regression **(A)**, signal inhomogeneity determined by Bayesian bias field correction **(B)**, and corrected M_0_ **(C)**.

A Qualitative proton density map was calculated for each subject in a voxel-wise manner by fitting the signal recovery curve to the mono-exponential equation *S* = *M_0_^∗^[1-e^(-TR/T_1_)]* to yield a map of M_0_ in arbitrary units. Next a Bayesian bias field correction (Figure 2) was applied to the M_0_ maps to account for inhomogeneity in the receiver coil profile (Iglesiast et al., 2016). The low spatial frequency bias field was calculated using 4th order polynomials and six Gaussian components. To avoid artificially attenuating the higher signal water phantoms, the blood and water phantoms were excluded when calculating the smooth bias field, and the correction was then applied to the entire volume (see Figures 1B,C).

The calculation of M_0_ values by voxel-wise exponential regression on the signal recovery curves resulted in a range of M_0_ values on an arbitrary scale. Due to the qualitative nature of MRI signal measurement, the average value of M_0_ in brain tissue varied from subject to subject on this arbitrary scale. Therefore, the M_0_ maps were calibrated to absolute water content in two ways. The first follows the previously published CaSTRR technique which uses a series of five phantoms containing mixtures of deuterium oxide and distilled water at 40, 30, 20, 10, and 0% (Thalman et al., 2019). These phantoms were also doped with 0.18 mM gadobutrol (Gadavist, Bayer Healthcare Pharmaceuticals, Whippany, NJ, United States) to reduce the longitudinal relaxation rate (T1) to be similar to the T1 of brain tissue (≈ 1.2 s). Because the deuterium oxide does not produce signal in MRI this creates a scale of known water concentration from 60–100% water. Each voxel in the image can then be normalized to this internal scale to yield a measure of absolute water content. It was noted, however, that in subjects where the arbitrary M_0_ values of brain tissue were high, the M_0_ values of the doped water phantoms tended to be higher than expected. This meant that while the M_0_ value of tissues increased linearly with the overall image intensity, the M_0_ values of the phantoms exhibited a quadratic increase over the range of arbitrary M_0_ values measured. The result was that the ratio of average M_0__,tissue_ to average M_0__,phantoms_ used in image normalization was not constant from subject to subject but had a strong negative linear relationship to the phantom signal intensity. To correct for the relationship, linear regression of this ratio against the average M_0_ value of the phantoms was used to quantify the quadratic error term for each subject. This negative error term was then subtracted from the blood and tissue M_0_ values prior to normalization. Finally, a linear regression was calculated based on the average M_0_ value in each phantom and its known water content, and every voxel in the image was normalized to the resultant equation (see Figure 1E).

The second method of calibrating M_0_ maps utilized the arterial Hct value to determine the absolute water content of the blood sample. Water content was determined according to the equation *WC_*blood*_* = *−0.271^∗^Hct* + *0.912* (Lijnema et al., 1993). The M_0_ image was then normalized such that the average water content in the blood sample matched the average value calculated according to hematocrit.

Brain–blood partition coefficient maps were generated for both normalization methods by comparing the measured water content of each voxel in the brain to the average water content of the blood using the equation *BBPC* = *WC_*brain*_/(WC_*blood*_^∗^1.04 g/mL)* (see Figure 1D).

### Cerebral Blood Flow and Anatomical Imaging

The CBF maps were acquired using a pCASL sequence with a three-dimensional gradient and spin echo (GRASE) readout (Alsop et al., 2015). The acquisition parameters were as follows: TR/TE/TI = 3200/16/1400 ms, field of view = 270 × 250 × 90 mm, matrix = 96 × 88, resolution = 3 × 3 × 3 mm, acquisition time = 6:15 min (see Figure 1F).

Because the original CBF maps were created with an assumed BBPC value of 0.9 mL/g, corrected maps were generated by dividing the entire map by this value and then multiplying by the BBPC map in a voxel-wise manner. BBPC correction was performed using the BBPC maps generated using the gadolinium doped water phantoms (see Figure 1G).

Contrast to noise ratio (CNR) was calculated according to the equation *CNR* = *(Mean_gray_ – Mean_white_)/Pooled Standard Deviation* (Cohen, 1988).

Anatomical images were acquired using a high resolution T1 weighted magnetization prepared rapid acquisition gradient echo (MPRAGE) sequence as recommended for optimal use with FreeSurfer (MGH, 2009). Scan parameters were: TR/TE = 1690/2.56 ms, flip angle = 12°, field of view = 320 × 320 × 160 mm, matrix = 256 × 256 × 128, resolution = 0.8 × 0.8 × 0.8 mm, acquisition time = 10:49 min.

### Image Analysis

All images were coregistered by first resampling the anatomical volumes to match the slice thickness of the CaSTRR and pCASL acquisitions. The CaSTRR and pCASL volumes were then registered to the anatomical using an intensity-based registration algorithm in Matlab (Mathworks, Natick, MA, United States) (Styner et al., 2000). The brain region of interest was extracted manually, and then segmented into gray and white regions of interest using an expectation maximization algorithm with classes for gray matter, white matter, and cerebrospinal fluid (Wells et al., 1996). To avoid partial volume effects, the gray and white matter regions of interest in each slice were eroded by two pixels. Due to image inhomogeneities in the MPRAGE acquisitions in some animals, the segmentation algorithm often failed to adequately segment gray and white matter regions in the most rostral and caudal sections of the brain. While the BBPC maps did not display these inhomogeneities, the regions of interest were not reliable for analysis. For this reason, we chose to include only the centermost 10 slices in our analysis. These regions of interest were then applied to the BBPC and CBF maps. All image analysis was performed in Matlab (Mathworks, Natick, MA, United States).

### Statistical Analysis

All statistical analysis was performed in Matlab. Data is expressed as mean ± standard deviation. Gray and white matter comparisons were assessed using three-way analysis of covariance with age, sex, and tissue type as independent variables. Linear regressions against age were also controlled for sex. Values of *p* < 0.05 were considered statistically significant.

## Results

### BBPC Is Higher in Gray Matter Than in White Matter

**FIGURE 3:**
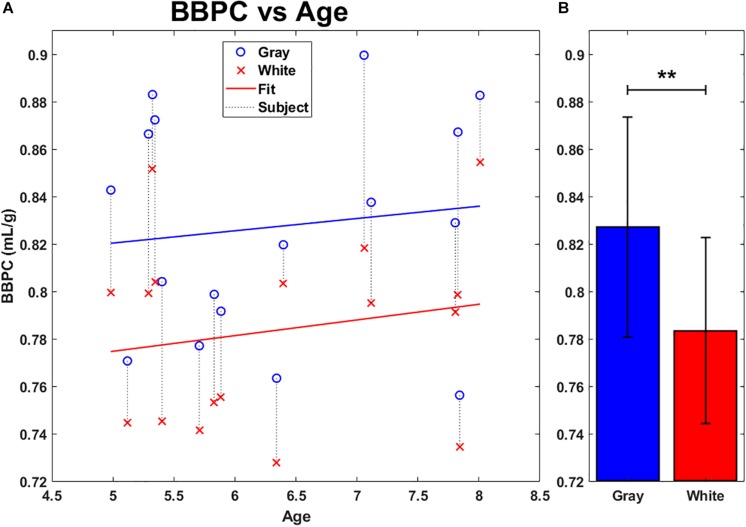
Average BBPC in gray matter (blue) and white matter (red) regions of interest plotted against age **(A)** and as group averages **(B)**. Regions corresponding to the same animal are connected with a vertical dotted line. No significant linear correlation to age was found in either region. Average BBPC in gray matter was which is 5.6% higher than in white matter (BBPC_*gray*_ = 0.83 ± 0.05 mL/g, BBPC_*white*_ 0.78 ± 0.04 mL/g, ^∗∗^ indicates p < 0.01, error bars represent 1 standard deviation).

The first comparison was drawn on BBPC maps generated using the previously published CaSTRR method of normalizing to the gadolinium doped water phantoms (see Figure 1E). The average BBPC in gray matter was 0.83 ± 0.05 mL/g which is 5.6% higher than the BBPC in white matter (0.78 ± 0.04 mL/g, *p* = 0.007) (see Figure 3B). When plotted against age, neither the gray nor the white matter regions of interest demonstrated a significant correlation over the age range studied (gray matter: *p* = 0.645, white matter: *p* = 0.483 (see Figure 3A).

### Gray Matter CBF Is Negatively Correlated With Age

**FIGURE 4:**
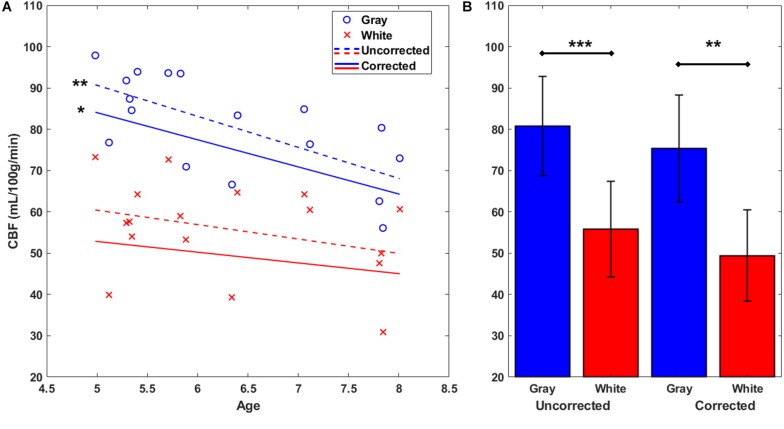
Gray and white matter perfusion plotted against age **(A)**. Plotted points represent uncorrected average CBF for each animal. Gray matter CBF demonstrates a negative linear correlation with age in both uncorrected CBF maps (CBF_*gray*_ = 128 – 7.5 ^∗^ Age mL/100 g/min) and maps corrected using measured BBPC (cCBF_*gray*_ = 117 – 6.6 ^∗^ Age mL/100 g/min). Linear regression was not significant in the white matter region in either case. When considered as group averages **(B)**, the gray matter has significantly higher CBF in both uncorrected (CBF_*gray*_ = 81 ± 12 mL/100 g/min, CBF_*white*_ = 56 ± 12 mL/100 g/min), and corrected maps (cCBF_*gray*_ = 73 ± 13 mL/100 g/min, cCBF_*white*_ = 49 ± 11 mL/100 g/min). ^∗^ indicates *p* < 0.05, ^∗∗^ indicates *p* < 0.01, ^∗∗∗^ indicates *p* < 0.001, error bars represent 1 standard deviation.

Next, gray and white matter perfusion were compared in uncorrected CBF maps (see Figure 1F) and again in corrected CBF maps (see Figure 1G) which used the standard CaSTRR derived BBPC maps for correction (see Figure 1E). Gray matter has 45% higher CBF than white matter in the uncorrected CBF maps (CBF_*gray*_ = 81 ± 12 mL/100g/min, CBF_*white*_ = 56 ± 12 mL/100 g/min, *p* < 0.001) and 53% higher CBF in the maps corrected for BBPC (cCBF_*gray*_ = 73 ± 13 mL/100 g/min, cCBF_*white*_ = 49 ± 11 mL/100 g/min, *p* < 0.01) (see Figure 4B). Gray matter demonstrated a significant negative correlation with age with a reduction of 7.5 ± 2.1 mL/100 g/min each year or 9% of the average perfusion (CBF_*gray*_ = 128 – 7.5 ^∗^ Age mL/100 g/min, *p* = 0.003). The corrected CBF maps also revealed a reduction of 6.6 ± 2.6 mL/100 g/min/year (cCBF_*gray*_ = 117 – 6.6 ^∗^ Age mL/100 g/min, *p* = 0.02), but this relationship was not significantly different in the corrected maps than the uncorrected (*p* = 0.81). While there appears to be a downward trend in white matter perfusion with age, this correlation was not statistically significant in the uncorrected CBF maps (*p* = 0.20) or the corrected maps (*p* = 0.33) (see Figure 4A).

### BBPC Correction Improved Contrast to Noise Ratio in CBF Maps

Next, the CNR of corrected CBF maps (see Figure 1G) was compared to uncorrected CBF maps (see Figure 1F). On average BBPC correction improved CNR between gray and white matter regions of the CBF maps by 3.6% (95% confidence interval = 0.6 – 6.5%). The average uncorrected CNR was 0.81 and the average corrected CNR was 0.84.

### BBPC Values Generated Using Hematocrit to Estimate Water Content Agree With Those Generated Using Water Phantoms

**FIGURE 5:**
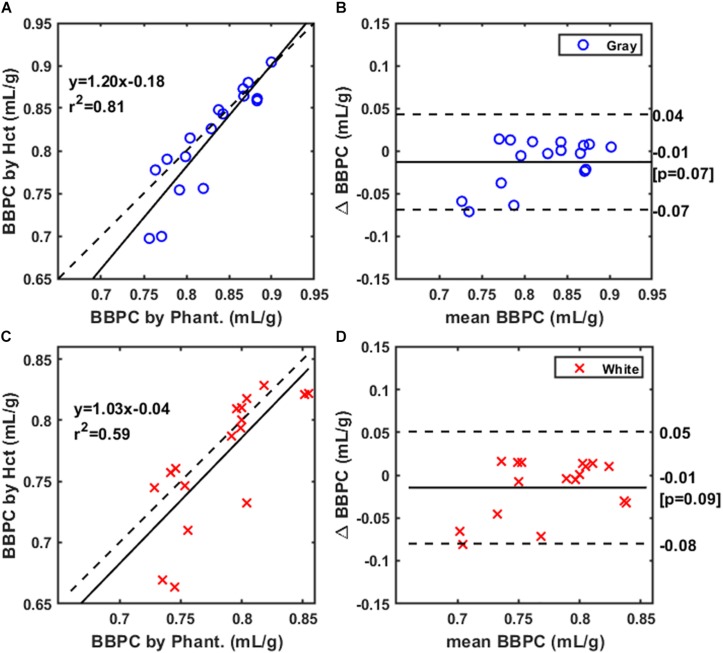
Correlation of BBPC values in maps normalized according to hematocrit (Hct) with values in maps normalized to the gadolinium-doped phantoms. Correlation is very strong for the gray matter BBPC values (*R*^2^ = 0.81) **(A)** and moderately strong for white matter (*R*^2^ = 0.59) **(C)**. Bland-Altman analysis demonstrates no significant bias in either gray **(B)** or white matter values **(D)**.

The final comparison was between maps normalized using the doped water phantoms (see Figure 1E) to ones normalized using Hct derived blood water content (see Figure 1D), we observed positive correlations between the BBPC values generated using these two methods. The Pearson correlation was *R*^2^ = 0.81 for gray matter indicating strong correlation between these measures in this region (see Figure 5A). Due to higher variability in the white matter regions the correlation was moderate in white matter (*R*^2^ = 0.59) (see Figure 5C). The measured BBPC values were slightly lower in maps normalized to hematocrit, though not statistically different, and Bland-Altman analysis demonstrates no significant bias in either region of interest (see Figures 5B,D). Again the BBPC in gray matter was 5.9% higher than in white matter when using Hct to normalize (BBPC_*gray*_ = 0.81 ± 0.06 mL/g, BBPC_*white*_ = 0.77 ± 0.05 mL/g, *p* = 0.02).

## Discussion

The CaSTRR technique represents a significant improvement in the acquisition speed of BBPC maps. A previously published report measuring BBPC using a 1.5T human scanner acquired a single slice of the BBPC map in approximately 30 min (Roberts et al., 1996). In this study we were able to produce BBPC maps of sufficient quality to perform voxel-wise correction of CBF maps with coverage of the entire brain using a CaSTRR acquisition protocol that only required 4 min of scan time. This is an improvement over the reported CaSTRR technique in mice which required 17 min of scan time due to the much higher resolution requirement of scanning small animals at 7T (Thalman et al., 2019). A 4 min scan time is comparable to the acquisition time of the pCASL technique for CBF. The experiment was also done with commercially available equipment and pulse sequences that are directly applicable for use with a human subject. Furthermore, the CaSTRR scans were performed with the same birdcage receive coil that was used for the pCASL acquisitions. This was not possible when scanning mice at 7T and represents a significant advantage of scanning at lower fields. The greatly reduced scan time and ready availability of equipment and pulse sequences demonstrate that CaSTRR is a feasible approach to correct CBF maps using empirically measured BBPC values instead of assuming a constant value for all tissue types, pathologies, ages, and species. This technique has the potential for rapid translation to use in human studies.

While BBPC has not been previously reported in canines, our reported values of 0.83 ± 0.05 mL/g in gray matter and 0.78 ± 0.04 mL/g in white matter are lower than published reports in humans, non-human primates, and mice (Herscovitch and Raichle, 1985; Kudomi et al., 2005; Leithner et al., 2010). One possible reason for this is the temperature discrepancy between the blood sample and the brain tissue. When measured at room temperature instead of physiologic temperature, the proton density of the blood could be overestimated by as much as 5% causing BBPC to be underestimated by the same amount (Tofts, 2003). The amount of inter-species variability in BBPC values is further evidence for the importance of empirical BBPC correction when quantifying CBF.

The correlation between the two methods of normalization represents a distinct advantage of this study. Our results suggest that the CaSTRR technique can be further simplified by omitting the gadolinium doped water phantoms. One of the difficulties of this study was the non-linear signal increase observed in the water phantoms. This is possibly due to the pre-scan normalization algorithm of the scanner used for this study and/or reduced T2^∗^ decay in the water phantoms. These effects would likely be specific to a given scanner and would need to be determined empirically. However, the correlation between BBPC values derived using the water phantoms with those derived using Hct indicate that future studies using CaSTRR could rely solely on the water content determined by the hematocrit. This would also obviate the need for correcting the non-linear signal increases observed in the water phantoms as done in this study.

Another significant advantage of this study is the use of Bayesian bias field correction to account for inhomogeneities in the receiver coil sensitivity profile. The CaSTRR method described in mice assumed a sufficiently homogenous profile in the birdcage receive coil, but observed significant signal loss near the large ear canals of the mouse (Thalman et al., 2019). Other BBPC studies attempted to correct for bias field using a separate measurement on a uniform phantom (Roberts et al., 1996; Leithner et al., 2010), but it is unlikely that the receiver profile would be the same when measuring the non-uniform tissue of a live subject.

Arterial spin labeling has an inherently low signal to noise ratio because it is a subtractive technique. Including an empirical measurement of BBPC to the quantification of CBF will increase noise, as we observed in the greater variance of CBF values in the corrected maps. However, there was an improvement in contrast to noise between areas with significantly different BBPC values despite this addition of noise. This could become important when studying perfusion in models of pathology where the tissue composition is likely to change.

Canines have been widely used for aging and neurodegeneration studies (Martin et al., 2011; Vite and Head, 2014; Triani et al., 2018). There are many examples of pathologies that could affect water balance in the brain. Brain edema caused by ischemia (Rosenberg, 1999), infection (Niemoller and Tauber, 1989), or trauma (Winkler et al., 2016) can cause localized increases in free water and potentially affect the BBPC. Another important field of interest where ASL is commonly used is the study of Alzheimer’s disease (AD). The deposition of hydrophobic amyloid-β protein, which is a hallmark of AD pathology, may reduce the BBPC in regions of plaque development (Aleksis et al., 2017). AD occurs in the context of aging and typically causes pronounced brain atrophy (Bobinski et al., 1999). Both of these could result in reduced brain water content and therefore reduced BBPC. Both brain volume (Duning et al., 2005) and Hct (Jimenez et al., 1999) can also change significantly with a subject’s level of hydration. So while our result showed that BBPC correction did not affect the observed relationship between CBF and age between the ages of 5–8 year, it is possible that BBPC correction could improve sensitivity in studies where BBPC is expected to change significantly. In future studies, CaSTRR imaging could be used to study how BBPC changes in canines with pathology and could also be used to account for water balance effects when measuring perfusion.

We acknowledge that in addition to CaSTRR, other efforts have also been made to improve the BBPC measurement, CBF quantification, and gray-white CBF contrast. It has been reported that a uniform, brain-tissue-type-dependent magnetization image could be generated using a sensitivity calibration (Dai et al., 2011). Another study showed that BBPC can be improved by exploiting the partial-volume data to adjust the ratio between BBPC and the proton density-weighted image (Ahlgren et al., 2018). Furthermore, gray-white matter CBF can be enhanced with background suppression methods (van Osch et al., 2009). Here we provide another approach that can quantify BBPC rapidly, and improve CBF quantification and gray-white CBF contrast using Hct calibration.

## Conclusion

In conclusion, this study demonstrates the feasibility of CaSTRR as a method to correct CBF measurements for regional and inter-subject variability in BBPC. Further, we demonstrated that the correction can be achieved using Hct calibration. The developed CaSTRR method has potential contribution for future translational studies in aging and neurodegeneration.

## Data Availability Statement

All datasets generated for this study are included in the article/Supplementary Material.

## Ethics Statement

The animal study was reviewed and approved by the Institutional Animal Care and Use Committee of the University of Kentucky.

## Author Contributions

ST was responsible for the experimental and scanning protocol design, analysis software development, image acquisition, data and statistical analyses, and manuscript preparations. DP contributed to the sequence development, scanning protocol design, technical support, and manuscript editing. MU was responsible for the animal handling, image acquisition, laboratory samples, and manuscript editing. CN and EH contributed to the project design, animal acquisition, data interpretation, and manuscript editing. A-LL was the primary investigator and contributed to the project design, interpretation of the results, and manuscript preparation.

## Conflict of Interest

The authors declare that the research was conducted in the absence of any commercial or financial relationships that could be construed as a potential conflict of interest.

## Supplementary Material

FIGURE S1 B_1_ mapping. The B_1_ mapping was aquired using a *Tfl_b1map* sequence with a saline phantom (salinity is the same as mammalian extracellular space). Right image proportional to flip angle, showing maximum flip angle variation of 4% over a 10 cm phantom. The FOV is comparible to beagle’s brain, which is approximately 4 cm × 5 cm × 7 cm. **(Left)** The magnitude image. **(Right)** A flip angle map where the intensity is linearly proportional to the flip angle. The examples are shown in two ROIs: 83.8 and 89.5 degrees, respectively.
