# Supplementary figures and images for: Brain–Blood Partition Coefficient and Cerebral Blood Flow in Canines Using Calibrated Short TR Recovery (CaSTRR) Correction Method
Source: Front Neurosci. 2019 Nov 5;13:1189. doi: 10.3389/fnins.2019.01189 (PMC6848028; doi:10.3389/fnins.2019.01189)

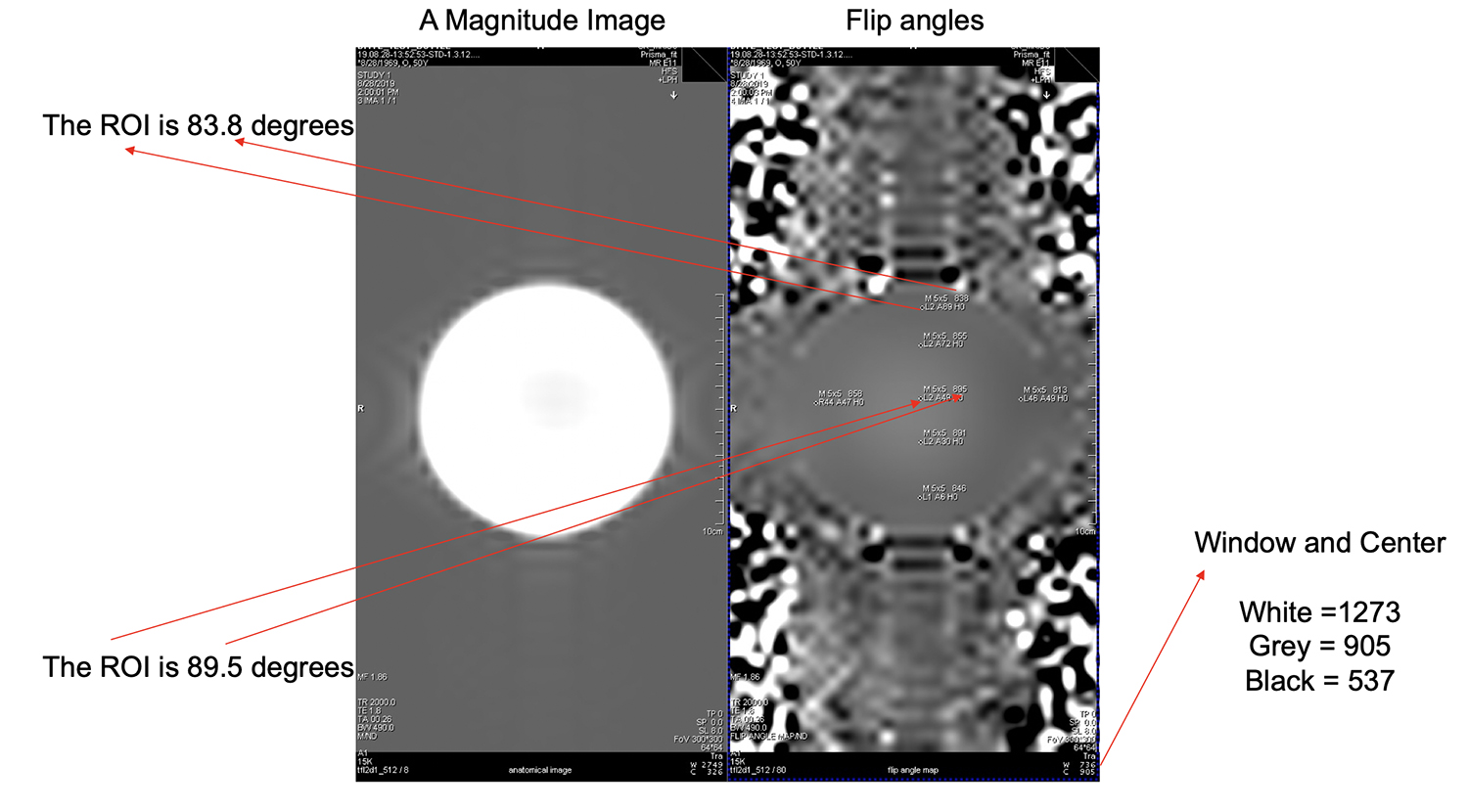

Supplement: FIGURE S1 — B1 mapping. The B1 mapping was aquired using a Tfl_b1map sequence with a saline phantom (salinity is the same as mammalian extracellular space). Right image proportional to flip angle, showing maximum flip angle variation of 4% over a 10 cm phantom. The FOV is comparible to beagle’s brain, which is approximately 4 cm × 5 cm × 7 cm. (Left) The magnitude image. (Right) A flip angle map where the intensity is linearly proportional to the flip angle. The examples are shown in two ROIs: 83.8 and 89.5 degrees, respectively. [file Image_1.JPEG]
